# Predator perception of aposematic and cryptic color morphs in two *Oophaga* species

**DOI:** 10.1002/ece3.70351

**Published:** 2024-09-30

**Authors:** Vasiliki Mantzana‐Oikonomaki, Ariel Rodríguez, Giselle Castillo‐Tamayo, Roberto Ibáñez, Heike Pröhl

**Affiliations:** ^1^ Stiftung Tierärztliche Hochschule Hannover Institut für Zoologie Hannover Germany; ^2^ Centro de Investigaciones en Productos Naturales (CIPRONA) & Escuela de Química Universidad de Costa Rica San José Costa Rica; ^3^ Smithsonian Tropical Research Institute Panama City Panama

**Keywords:** conspicuousness, *Oophaga*, predator avoidance, visual contrasts, visual modeling

## Abstract

Animals that are toxic often advertise their unprofitability to potential predators through bright aposematic colors while cryptic ones blend in with their natural background to avoid predators. In the poison dart frogs, *Oophaga pumilio* and *O. granulifera,* some populations in Costa Rica and Panama display cryptic green and aposematic red color morphs. We herein used reflectance spectra from the dorsum of red and green morphs of these frogs to estimate their perception by the visual systems of three potential predators (birds, lizards, and crabs) against three natural backgrounds (leaves, trunks and leaf litter). Statistical analyses revealed no strong differences in color contrast against backgrounds between the two frog species. However, strong effects of frog morph, predator, background, and their interactions were observed. When viewed against diverse backgrounds, red frogs of both *Oophaga* species are more color conspicuous to birds and Anoline lizards than to crabs. A strong effect of species was observed on luminance contrast. Concerning the latter, green frogs particularly in *O. granulifera* appear more conspicuous than red frogs, while birds perceive higher brightness contrasts than lizards or crabs. Our results further support the importance of birds and lizards as *Oophaga* predators and provide a first quantitative comparison of conspicuousness between these two frog species.

## Graphical abstract

In this manuscript, we investigate color variances between aposematic and cryptic color morphs of two *Oophaga* species and under the visual system of three different predators. No significant variations was observed in color and luminance contrasts between species.

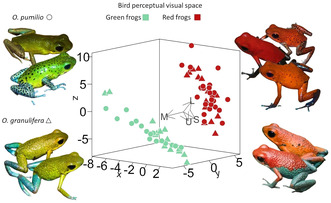

## 1 INTRODUCTION

Phenotypic variation among and within populations of species has been at the center of attention of evolutionary biologists trying to unravel the selection dynamics that promote divergence (Bull, 1987; Roulin, 2004). Animal coloration is a phenotypic trait with high significance for signaling and communication (Cuthill et al., 2017), allowing scientists to understand these selection processes. Aposematic, brightly colored animals advertise their toxicity or unpalatability to potential predators (Poulton, 1890) and predator selection favors convergence in such warning signals since they will be associated with the prey's noxiousness by experienced predators (Summers, 2003). Contrastingly, animals without chemical defenses usually utilize cryptic colorations to avoid predation and blend in with their surroundings (Ruxton et al., 2004). If cryptic or aposematic, color and patterns can vary among different allopatric populations of the same species, as observed in polytypism (Mayr, 1963), or sympatrically within populations, as in polymorphic species (Ruxton et al., 2004).

A warning signal may vary depending on the predator's position, as it might appear cryptic from afar but aposematic up close (Barnett et al., 2016; Barnett & Cuthill, 2014). Variation in predation avoidance mechanisms is further promoted by the predator's ability to perceive color signals (Osorio & Vorobyev, 2008). Visually oriented predators differ significantly in their abilities to perceive warning signals, with some predators detecting signals that others do not because of differences in their visual systems (Osorio & Vorobyev, 2008). In addition, for any given predator, chemically defended prey vary in the degree of unprofitability, as for some it could be simply distasteful while for others, it could be a source of deadly toxins (Mappes et al., 2005). Further differences in sensory processing such as detectability, discriminability, and willingness to attack, also play a role in warning signal effectiveness (Guilford & Stamp, 1991). Warning coloration therefore varies on effectiveness among predator communities which may lead to conflicting selective pressures resulting in aposematic or cryptic coloration (Briolat et al., 2019).

Aposematic theory predicts that high levels of warning signal diversity will not be promoted as that would increase the costs of “educating” predators about their unpalatability (Endler, 1988; Ruxton et al., 2004). However, diversification in coloration and warning signals does occur in polytypic species (Briolat et al., 2019). These differences can sometimes be explained by geographic differences in predation pressures (Chouteau & Angers, 2012; Mallet & Barton, 1989; Noonan & Comeault, 2009) or the interaction between predation and other selection forces like mate selection (Cooney et al., 2019).

The monophyletic family of Neotropical poison frogs Dendrobatidae is a well‐known system for studying color diversity and the relationships among coloration and defensive strategies (Summers & Clough, 2001). For example, the poison frog *Ranitomeya imitator* (Schulte, 1986) mimics the aposematic coloration of sympatric dendrobatid frogs (Symula et al., 2001). The common warning signal is then promoted through Müllerian mimicry (Chatelain et al., 2023). Furthermore, phylogenetic analyses of dendrobatid frogs have shown the joint evolution of aposematic traits, including conspicuousness, chemical defenses, diet specialization, and high metabolic rate, leading to the “aposematic phenotype” in several lineages (Santos & Cannatella, 2011; Saporito et al., 2004, 2006; Saporito, Zuercher, et al., 2007).

One member of this family, the strawberry poison frog, *Oophaga pumilio* (Schmidt, 1857), ranges from Nicaragua to Panama. Throughout most of its range, it displays a reddish‐orange dorsum with blue or dark hind legs. In the Bocas del Toro Archipelago of Panamá, however, this species shows a high diversity of coloration (Rudh et al., 2007; Saporito, Zuercher, et al., 2007) including more aposematic (e.g. red or orange) and more cryptic morphotypes (e.g. green or blue) among populations (Dreher et al., 2015). Another species of the same genus, *Oophaga granulifera* (Taylor, 1958) displays a dorsal color gradient from green morphotypes in the north to red morphotypes in the south across its range in Costa Rica (Brusa et al., 2013). Red and green morphotypes are found at the geographic extremes, with orange and brown intermediates in between.

Using visual modeling, it has been demonstrated that the most conspicuous morphotype of *O. pumilio* for predatory birds was a red‐bodied morph present in the mainland Panamá, in the Bocas del Toro region (Siddiqi et al., 2004). Similarly, red *O. granulifera* are considered to be more aposematic than green ones in terms of detectability (Willink et al., 2013). Red coloration is expected to be more aposematic in general, as red colors (~640–700 nm) are the most conspicuous in tropical forests where both *O. pumilio* and *O. granulifera* are present (Alves‐Costa & Lopes, 2001; Endler, 1993; Osorio & Vorobyev, 2005; Wheelwright & Janson, 1985). In *O. pumilio*, conspicuousness for birds is correlated with toxicity, showing that coloration is an honest signal of unpalatability, i.e. the conspicuous red and orange frogs are the most toxic (Maan & Cummings, 2012). It has been revealed that *O. granulifera* toxicity follows the opposite pattern than in *O. pumilio*, as green morphotypes are more toxic than red morphotypes (Wang, 2011). In dendrobatid frogs, toxicity is related to alkaloids which the frogs acquire with their insect diet (Bolton et al., 2017).

Birds have been demonstrated to predate upon *Oophaga* frogs and are believed to influence selection towards the aposematic signal (Maan & Cummings, 2012). Specifically, the rufous motmot (*Baryphthengus martii*, Spix, 1824) has been documented as a predator (Master, 1998). Experiments with clay models indicate lizards, snakes and crabs as important predators as well (Dreher et al., 2015; Willink et al., 2014). *Anolis* lizards share habitats with *Oophaga* species across Panama and Costa Rica (Savage & Talbot, 1978). Land crab species like the blue land crab (*Cardisoma guanhumi*, Berthold, 1827) and the Neotropical land crab genus *Gecarcinus*, inhabit forests near the Coastal areas in the Atlantic and Pacific coast of the Neotropical realm and experimental evidence of crab predation on *Oophaga* clay models has established land crabs as potential *Oophaga* predators (Dreher et al., 2015).

In both *O. granulifera* and *O. pumilio*, the conspicuous red morphotype has been suggested to be the ancestral morph while other morphotypes have diverged later. The color diversification between northern and southern *O. granulifera* lineages has been a recent evolutionary event (Brusa et al., 2013). In *O. pumilio*, the extreme color polymorphism in Bocas del Toro has recently diverged from the widespread mainland red and blue‐legged morphotype resulting from the separation of the mainland and islands (Brown et al., 2010).

Both these species offer a great system to study color divergence between more conspicuous and cryptic coloration throughout their geographic distribution. Color divergence is expected to be related to geographic variation of predator composition, because of predator‐driven divergence (Nokelainen et al., 2014; Willink et al., 2014). While the two species do not co‐occur, they share a similar evolutionary history and *O. granulifera* belongs to a sister lineage of the clade containing *O. pumilio* (Monteiro et al., 2023; Posso‐Terranova & Andrés, 2016). In addition, their contrasting patterns of toxicity puts them on the spot for exploring predator‐driven color divergence of toxic prey.

Several studies have investigated conspicuousness differences among populations of *O. pumilio* (Dreher et al., 2015; Maan & Cummings, 2012; Siddiqi et al., 2004) and among populations of *O. granulifera* (Willink et al., 2014). In this first comparative study between the two species, we compare the predator perception of diverging color morphs by examining the conspicuousness of the red and green morphotypes of two *Oophaga* species under the visual systems of three potential predators. We used spectrometric data of red and green frogs from different localities in Costa Rica and Panama and calculated differences in predator perception between frogs of all different localities and color and luminance contrasts between frogs and the common natural background. We expected to find a higher conspicuousness for red frogs in natural forest background and that predators can perceive visual differences in conspicuousness between red and green frogs in both species, but not between species.

## 2 METHODS

### 2.1 Sampling

**FIGURE 1:**
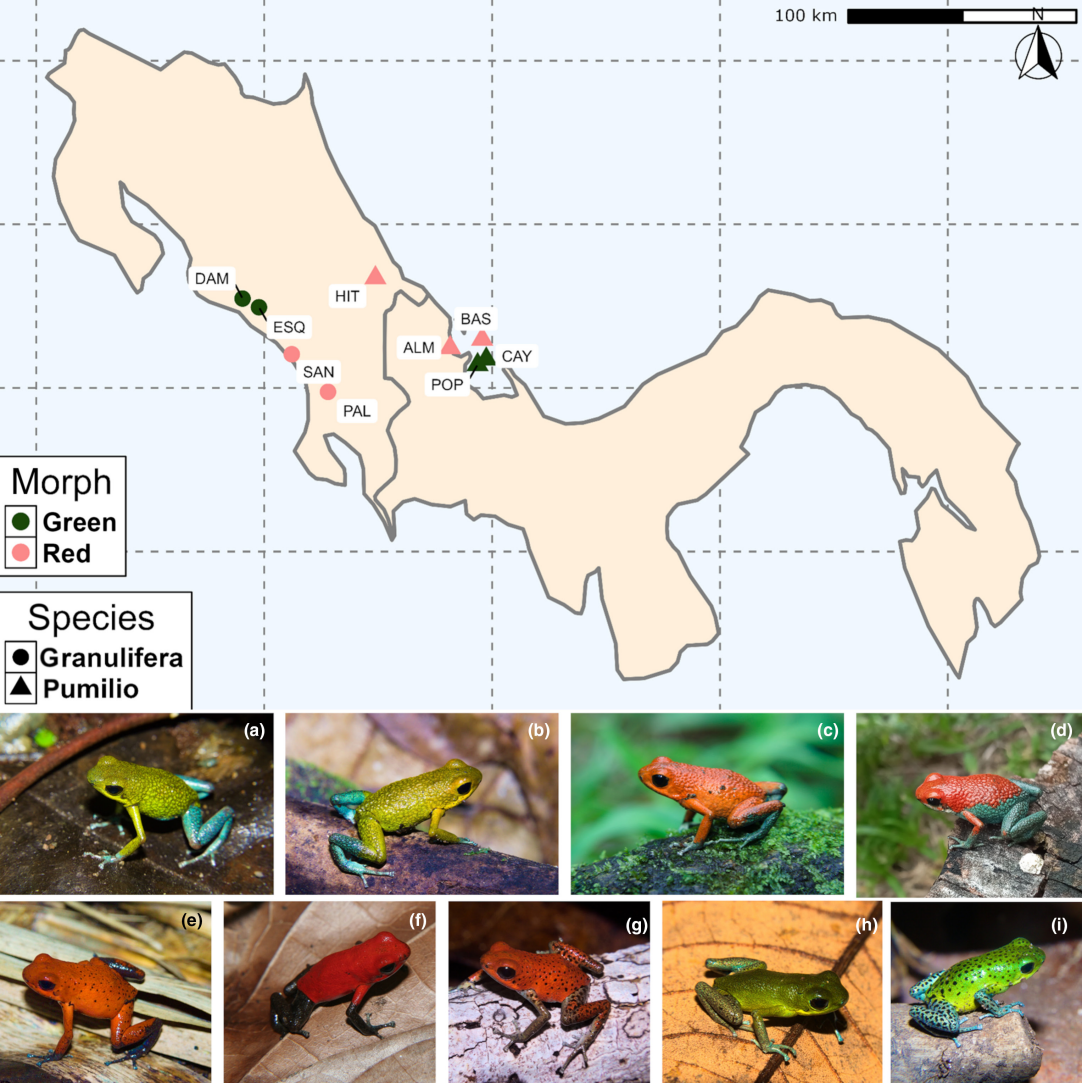
Map of sampling localities in Costa Rica and Panama for *Oophaga granulifera* (triangles) and *O. pumilio* (circles). The color of the symbol refers to the color of the frogs in each sampling locality (red: Red frogs, green: Green frogs). The first row of pictures refers to *O. granulifera* and the second to *O. pumilio* and the order follows the localities on the map from west to east and north to south. All collected frogs were males. (a) San Antonio de Damas (DAM) (b) Esquipulas de Quepos (ESQ). (c) Dominical in San Josecito (SAN). (d) Palmar Norte (PAL). (e) Hitoy‐Cerere (HIT). (f) Almirante (ALM). (g) Isla Bastimentos (BAS). (h) Cayo de Agua (CAY). (i) Isla Popa (POP). Photographs taken by ©AR and D, by © Abel Mora‐Machado.

During the rainy season in May–June 2022, we sampled red and green frogs of *O. granulifera* and *O. pumilio* frogs in Costa Rica and Panama (Figure 1). *Oophaga granulifera* is distributed along southwestern Costa Rica and is less common compared to *O. pumilio*. Possible sampling localities of *O. granulifera* are limited, and the more differentiated red morphotypes of the southern distribution and green morphotypes of the northern distribution range were sampled in Costa Rica. To compare conspicuousness of similar color morphs between the species, red and green morphotypes of *O. pumilio* were sampled in Panama in the Bocas del Toro Archipelago. An additional mainland population with red frogs was sampled in Costa Rica. We found large populations of hundreds of frogs and eight males were sampled at each locality. Since sexual dimorphism in color is absent in both species only males were sampled. Females are the limited sex in reproduction (Pröhl & Hödl, 1999), therefore it is less invasive to work with males at the population level. We used playback calls to attract males when needed and captured frogs were kept individually in plastic containers together with moist substrate until sampling was completed. Frogs were then transferred to the field station, where spectrometric and morphological measurements were taken within 2 h of capture.

In Costa Rica, we sampled a population of green *O. granulifera* at Hacienda Mil Bellezas (abbreviation: DAM) in San Antonio de Damas, Punta Arenas (N 9.54°, W 84.18°, elevation: 144 m, Figure 1a) and another one at Esquipulas de Quepos, Punta Arenas (abbreviation: ESQ) (N 9.49°, W 84.046°, elevation: 225 m, Figure 1b). In DAM, individuals were green on the dorsal side with light blue limbs. We observed a similar coloration in the frogs at ESQ with a light blueish coloration only at the outer end of their legs. Red frogs were sampled at Dominical in San Josecito, Punta Arenas (abbreviation: SAN) (N 9.20°, W 83.75°, elevation: 241 m, Figure 1c) and at Palmar Norte, Punta Arenas (abbrevations: PAL) (N 8.97°, W 83.43°, elevation: 365 m, Figure 1d). Frogs collected at SAN were bright red on all their dorsal area with teal green‐blue limb ends. In PAL, frogs were bright red on their central dorsal area with teal green‐blue sides and limbs.

One *O. pumilio* population was sampled at the Reserva Hitoy‐Cerere, Limon Province in Costa Rica (abbreviation: HIT) (N 9.67°, W 83.02°, Figure 1e). Male individuals in this locality were red in all the dorsal area with small dark dots and blue end legs. In the Bocas Del Toro archipelago one population with red *O. pumilio* was sampled on the mainland at Valle Agua Arriba, Almirante, Bocas del Toro (abbreviation: ALM) (N 9.24°, W 82.08°, Figure 1f) and one at Punta Vieja, Isla Bastimentos, (abbreviation: BAS) (N 9.29°, W 82.36°, Figure 1g). Male individuals collected at BAS, were deep red with dark dots on the dorsal area and red‐brownish limbs with dark dotted patterns. Individuals collected in ALM, were deep red on the dorsal area with dark limbs. Green frogs were collected on Cayo de Agua, (abbreviation: CAY) (N 9.17°, W 82.05°, Figure 1h) and on Isla Popa (abbreviation: POP) (N 9.14°, W 82.12°, Figure 1i). At CAY, frogs were dark green dorsally with blueish feet. On POP, male individuals were bright green with dark dotted pattern on the dorsal side and light blue‐colored limbs. Additionally, we collected different types of substrates found on the ground of each locality to represent the substrate types most frequently used by frogs during sampling. Posteriorly, we classified the substrates into categories and focused our analyses on three different types, which prevailed across all localities: leaf litter, tree trunks, and green leaves.

### 2.2 Spectrometry

Spectral reflectance (*R*) of the dorsal skin of captured males and substrates were measured with an Ocean Optics bifurcal optic fiber R‐200‐2‐UV/Vis connected to an Ocean Optics HR2000 spectrometer (Ocean Optics Inc., Dunedin, FL, USA). We used a deuterium‐tungsten DT‐Mini‐2‐GS lamp (Ocean Optics Inc.) as a light source and the spectrometer was calibrated with a WS‐1‐SS white standard (Ocean Optics Inc.) prior measuring each individual. Obtained reflectance spectra were exported using the OceanView 2.0.10. Boxcar width and spectra scans to average were set to 5, and integration time was set to 1000 ms. We took all reflectance measurements 1 to 2 h after field sampling, in a dark room, positioning the spectrometer probe 2 mm directly above the measured surface using a probe adapter. We measured four different points of the dorsum of each frog distributed in an anterior–posterior zig‐zag pattern. Six repeated measurements were taken from each substrate object. Obtained reflectance spectra were later post processed using the R package pavo (Maia et al., 2019). This included trimming the spectra to include measurements between 300 and 700 nm and setting the negative values representing noise to zero. To further remove spectral measurement noise, we smoothed the spectra using a LOESS function with a span of 0.2 and averaged the spectra of the four measurements taken per each individual to a mean value. The same pre‐processing steps were conducted for the substrate measurements, which, in the later step, were aggregated by type. Data provided following the guide recommended by White et al. (2015).

### 2.3 Visual modeling

To quantify the conspicuousness of the collected frogs, we evaluated total luminance by obtaining the total reflectance flux, following Endler (1990), and implemented viewer‐dependent and taxon‐specific detection models for potential frog predators, including a tetrachromatic UV sensitive bird, a tetrachromatic UV sensitive lizard and a dichromatic non UV sensitive crab. These three species were selected based on the existing information on *Oophaga* frogs' natural predators from predation experiments with plasticine models and scarce observations (supporting information S1 in Dreher et al., 2015; Santos & Cannatella, 2011). So far, few studies have succeeded in identifying natural predators of *Oophaga* at species level, however, predation studies identified marks on clay models from birds, lizards and land crabs (Dreher et al., 2015; Willink et al., 2014).

In all models, relative quantum catches at each photoreceptor were calculated from the spectra and for each frog. Differentiation between red and green frogs was evaluated by representation of the frogs in a viewer‐specific color space. The conspicuousness of the frogs was then evaluated in terms of color (Δ*S*) and luminance (Δ*L*) contrast relative to an estimated average background substrate of three different substrate types. Each model considers the reflectance spectra collected per frog and taxon‐specific photoreceptor absorbance spectra for each photoreceptor class of three different viewers. For irradiance, we used a standard irradiance measurement of forest shade data available in the pavo R package.

We first implemented a tetrachromatic UV‐sensitive visual system representing an average avian UV system using “avg.uv” in the “vismodel” command of pavo (Maia et al., 2019), following the model described in Vorobyev and Osorio (1998) (Figure 3a). The avian average UV system was selected to simulate the visual system of a potential bird predator. Short wavelength photoreceptors peak absorbance is at 370 nm (UVS, ultraviolet sensitive) and 458 nm (SWS, short wavelength sensitive). Medium wavelength photoreceptors (MWS, middle wavelength sensitive) have a peak absorbance at 547 nm and long wavelength photoreceptors at 600 nm (LWS, long wavelength sensitive). Luminance receptor stimulation was calculated based on the double cones as described for the blue tit *Cyanistes caeruleus* with a peak absorption at 563 nm (Hart et al., 2000). Quantum catches at each photoreceptor were calculated using “vismodel” in pavo, followed by calculating noise‐weighted Euclidian distances using “coldist” between the frogs and between the frogs and each of the three substrate types based on the receptor‐noise model of Vorobyev et al. (1998), Maia et al. (2019) and R Core Team (2023). Visual Weber fractions represent the noise to signal ratio in each photoreceptor and are independent of the intensity of the signal. In the long‐wavelength sensitive cone (LWS) it is calculated at 0.1 (Vorobyev et al., 1998) and at 0.05 for each of the double cones (Vorobyev et al., 1998). Relative photoreceptor abundances were 1, 1.9, 2.7, 2.7 for the avian model, following Hart et al. (2000). The UVS avian visual system was preferred over a non‐UV sensitive system to capture information from potential reflectance in the UV range.

For the second detection model, we used a tetrachromatic UV‐sensitive lizard visual system as described for *Anolis cristatellus* (Fleishman et al., 1997). The visual system of this species is similar in physiology and anatomy to those of other Anoline species studied (Fleishman et al., 1995, 1997; Loew et al., 2002; Persons et al., 1999). Anoline visual systems are adapted for high‐acuity diurnal vision and the retina contains four classes of cones modified by oil droplets (Fleishman et al., 1995). Spectral sensitivity for UVS of *A. cristatellus* peaks at 370 nm and SWS peaks at 495 nm. The peak sensitivity of the MWS is at 550 nm and for LWS at 590 nm (Fleishman et al., 2020). Oil droplet cut off was estimated at 330 nm for UVS, 371 nm for SWS, 463 nm for MWS and 507 nm for LWS (Loew et al., 2002). Luminance receptor stimulation was estimated based on the summed response of the two longest‐wavelength photoreceptors (Fleishman et al., 2020; Loew et al., 2002). Photoreceptor densities were set at 1:1:1:3 (Fleishman et al., 2020). Weber fractions for color and achromatic contrasts were modeled as described in Pérez I de Lanuza et al. (2018) and set at 0.05.

Finally, for the third detection model, we implemented a dichromatic visual system with no UV sensitivity for a crab predator, based on LWS cone absorbance spectra and electrophysiological measures of a short‐wavelength‐sensitive (SWS) cone response for *Uca thayeri* (Horch et al., 2002). In this model, SWS are most sensitive at 430 nm and LWS at 590 nm. Photoreceptor densities for the crab are estimated at 0.45 and 0.9 (Horch et al., 2002) and were set to 1 and 2 accordingly to estimate relative photoreceptor absorbance. Luminance receptor stimulation was estimated based on the summed response of both photoreceptors. Weber fraction was set to 0.12 based on information for single LWS cells of another arthropod, the honeybee, *Apis mellifera*, as such information is not available for the crab (Ibarra et al., 2000; Peitsch et al., 1992). A similar approach has been used in a previous study including visual modeling (Maan & Cummings, 2012).

Differences in how predators perceive red and green frogs were evaluated by calculating color distances between red and green frogs (coldist command in pavo), according to the receptor‐noise model of Vorobyev et al. (1998) with noise based on relative photoreceptor densities. Color distances were then transformed to Cartesian coordinates (jnd2xyz in pavo) and represented as corrected Cartesian coordinates in the perceptual color spaces of each of the predators modeled here.

Conspicuousness was evaluated in terms of color and luminance contrast between frogs and natural substrate. Contrasts were defined as the chromatic distance between two colors in units of just noticeable differences (JND), where 1 JND represents the minimum distance between two colors to be just barely discriminable (Vorobyev et al., 2001). To calculate visual contrasts between frogs and their natural background, the spectra of the substrates of the same category from the different localities were averaged and color distances between frogs and background were estimated in terms of color contrast and luminance contrast. In localities were color contrasts and luminance contrasts deviated between individuals, repeated measurements of the frogs were evaluated and spectral measurements which appeared to have more spectral noise were discarded. In addition, individuals which seemed to deviate greatly from the mean were considered outliers and were not included in the mean contrast estimations. All codes, raw data and outputs related to the visual modeling part can be found as a github repository provided at the data availability statement section.

### 2.4 Statistical analyses

We followed an information‐theoretic approach to analyze the relationship between the predictors and the two conspicuousness response variables: color (Δ*S*) and luminance contrast (Δ*L*). Information‐theoretic methods outperform null hypothesis testing whenever several alternative hypotheses can be drawn to explain variation, as typical in ecological and behavioral studies (Burnham et al., 2011; Mazerolle, 2006). In separated analyses, we fitted linear mixed effects models with individual as a random factor (to account for the replicated measurements per individual) and frog species, morph, substrate and predator (and their interactions) as predictors of Δ*S* and Δ*L* variation. The models were fitted with the function lmer from the lme4‐package in R (R Software Team, 2023). A total of 100 models for Δ*S* and 100 more for Δ*L* were fitted and we tabulated for each model the Akaike weight, ΔAICc, and evidence ratios (ERs) to represent selection uncertainty. In a second step, the relative importance of predictors, regression coefficients (*β*) and their associated 95% confidence intervals (CI) were calculated using a multi‐model inference approach. We then selected the models with Akaike weight > 0.01 (i.e. 95% confidence of containing the best fitting model) for the model‐averaged calculations and considered relevant those predictors with importance values above an 80%, a level which minimizes the probability of type I and II errors in simulations with sample sizes comparable with ours (Calcagno & de Mazancourt, 2010). Predictor effect was considered strong when its estimate CI excluded zero. Calculations were conducted using the glmulti package (Calcagno & de Mazancourt, 2010) in R. Model coefficients were estimated by multi‐model averaging of the top set of models summing up 95% of the evidence weight. Estimated coefficients were used to find the effect size of each fixed effect to the estimated variable, Δ*S* and Δ*L*.

## 3 RESULTS

### 3.1 Viewer‐specific distances between red and green frogs

**FIGURE 2:**
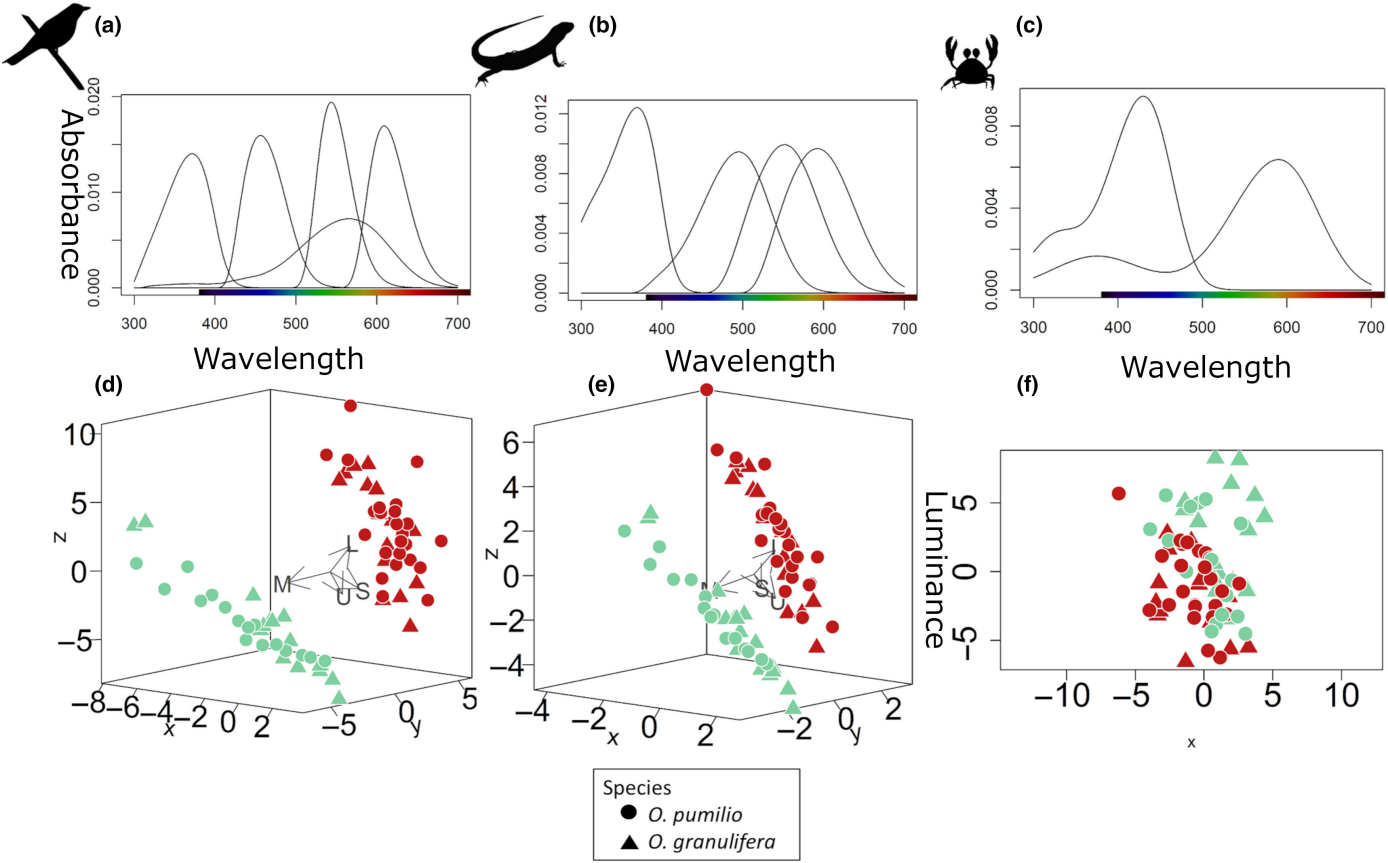
Models of spectral absorptions in the retina of the three potential predators ((a)—bird, (b)—lizard, (c)—crab) and perceptual color space representing color distances between *Oophaga granulifera* and *O. pumilio* frogs as estimated under the visual system of each of the three predators using relative quantum catch information. (d) Position of the studied frogs in the perceptual space of a model avian predator, as estimated from the matrix of inter‐individual color and luminance distances, plotted in arbitrary units. Receptor vectors are indicated with arrows (U – short wavelength receptor in UV, S – short wavelength receptor, M – medium wavelength receptor, L – long wavelength receptor). (e) Perceptual color space of an *Anolis cristatelus* lizard predator. (f) Perceptual color space graph of an *Uca thareyi* crab predator. The achromatic variable (luminance) is plotted on *y*‐axis to visualize the points in a two‐dimensional plot and was estimated as the summed response of the two photoreceptors of the crab visual system.

**FIGURE A1:**
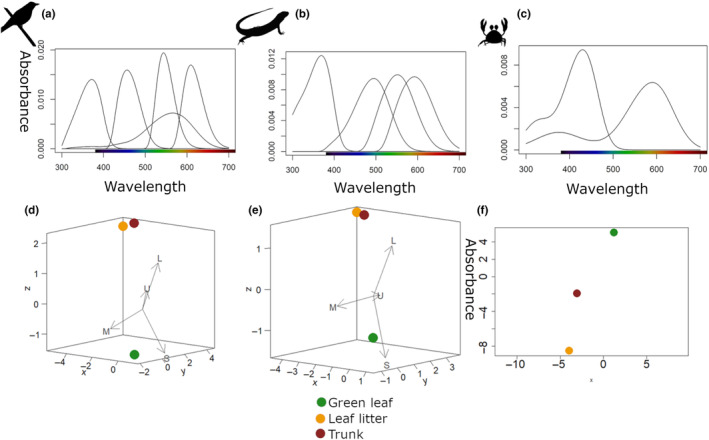
Models of spectral absorptions in the retina of the three potential predators ((a)—bird, –(b)—lizard, –(c)—crab) and perceptual color space representing color distances between the three substrate types as estimated under the visual system of each of the three predators using relative quantum catch information ((d)—bird, (e)—lizard, (f)–crab). Absolute position of the points in XYZ space is arbitrary. Receptor vector arrows (U – short wavelength receptor in UV, S – short wavelength receptor, M – medium wavelength receptor, L – long wavelength absorbance photoreceptor) show the direction of wavelengths in the color space. (d) Color space of an avian predator. Each substrate is shown as a different color shown on the legend. (e) Perceptual color space of an *Anolis cristatelus* lizard predator. (f) Perceptual color space graph of an *Uca thareyi* crab predator. The achromatic variable (luminance) is plotted on y axis to visualize the points in a two‐dimensional plot and was estimated as the summed response of the two photoreceptors of the crab visual system.

Perceptual visual spaces show how differently the red and green frogs are perceived by the three predators (Figure 2). Under the visual system of the avian predator, red and green frogs occupy distant and opposite positions in perceptual space (Figure 2d). Green frogs of both species largely overlap in their positions in perceptual space and the same is true for red frogs of both species. Under the visual system of *A. cristatelus*, we observe a similar pattern with no obvious separation between species (Figure 2e). Under the crab visual system red and green frogs occupy all positions of the *x*‐axis in the color graph (Figure 2f). Red and green frogs appear to occupy the opposite ends of the color space with a large overlap between species and morphs in the center of perceptual space. In the crab color space, the *x*‐axis is representing the direction of long wavelength to the left and short wavelength receptors to the right. For representing the points in the crab perceptual color space, the *y* axis was added to represent luminance, estimated as the summed response of the two photoreceptors. The average spectra of each type of substrate used to calculate color and luminance contrast of frogs against substrate is shown in the perceptual spaces of the three predators for reference (Appendix 1: Figure A1). Leaf litter and trunk are closer in the bird and lizard perceptual spaces whereas green leaf is more differentiated (Appendix 1: Figure A1d,e). In the crab perceptual space, the three substrate types appear distant to each other (Appendix 1: Figure A1f).

**FIGURE 3:**
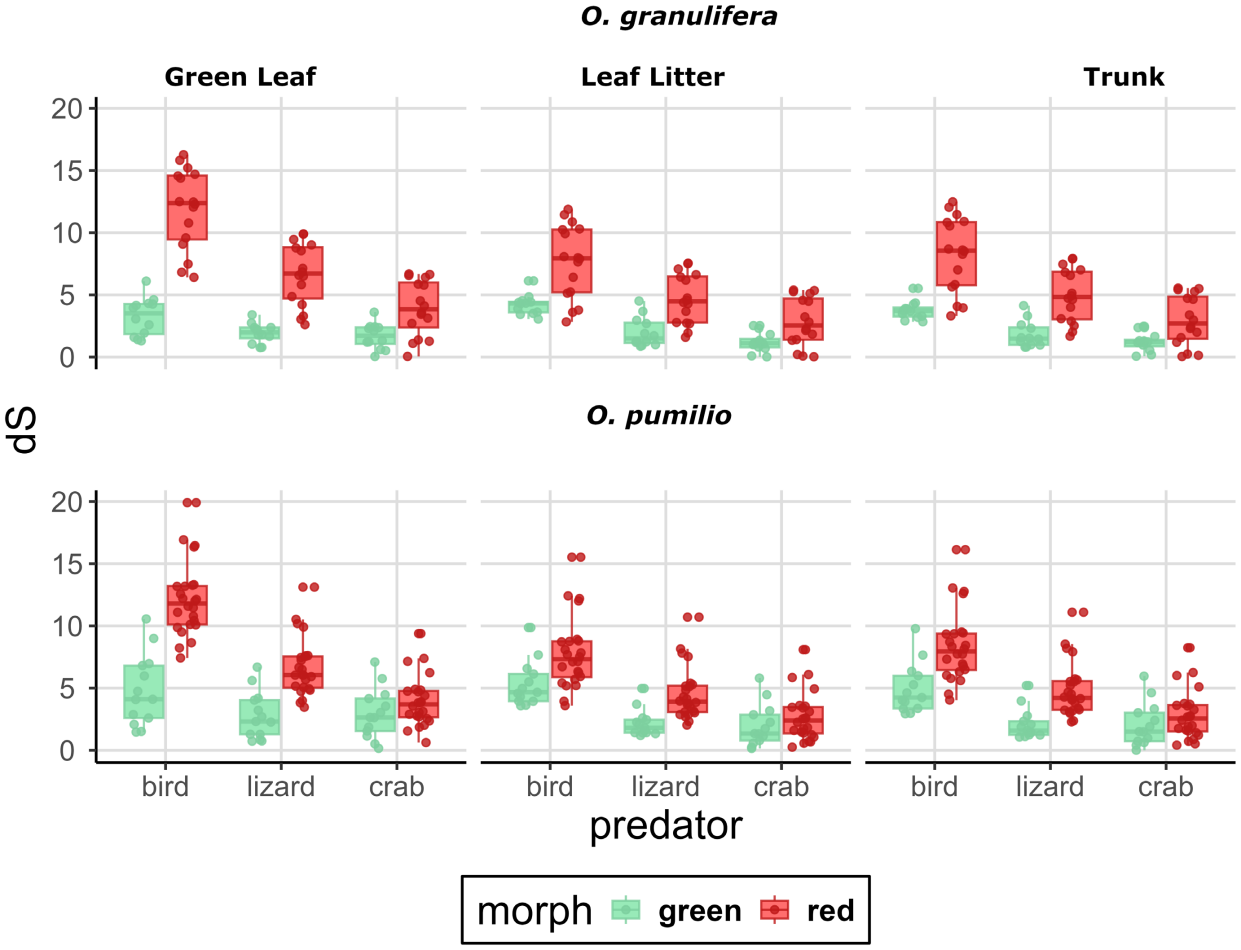
Each boxplot represents the distribution of Δ*S* (color contrast) measured for red and green *Oophaga granulifera* and *O. pumilio* frogs. Panel rows display the two species (top: *O. granulifera*, bottom: *O. pumilio*). The whiskers extend to the minimum and maximum values. The line within each box indicates the median Δ*S* value for that group. Each column displays a substrate type (from left to right: Green leaf, leaf litter, and trunk. Each predator is represented on the *x*‐axis in following order: Bird, lizard, crab, and colors represent the morphotype as indicated on the legend.

**FIGURE 4:**
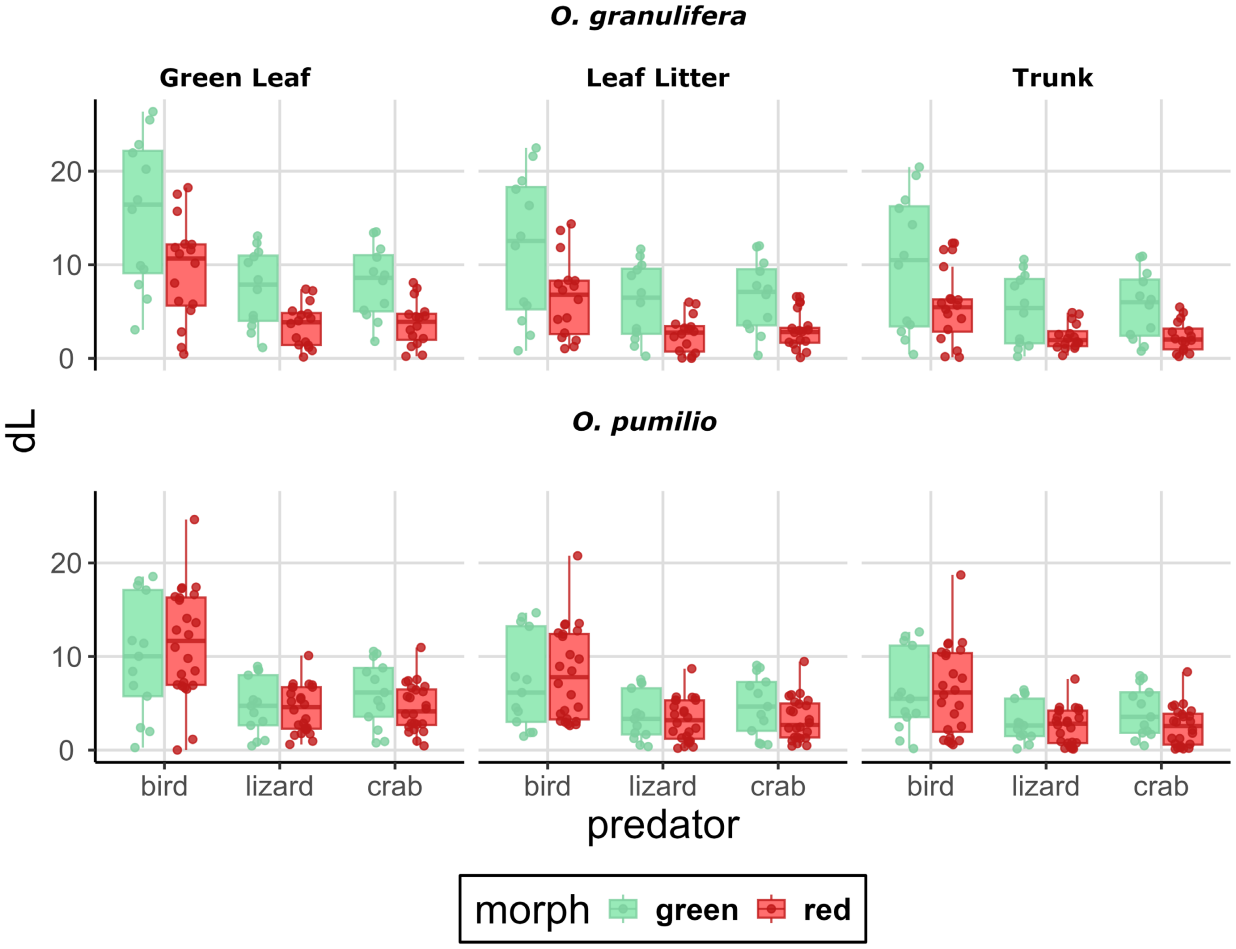
Each boxplot represents the distribution of Δ*L* (Luminance contrast) measured for red and green *Oophaga granulifera* and *O. pumilio* frogs. Panel rows display the two species (top: *O. granulifera*, bottom: *O. pumilio*). The whiskers extend to the minimum and maximum values. The line within each box indicates the median Δ*L* value for that group. Each column displays a substrate type (from left to right: Green leaf, leaf litter and trunk. Each predator is represented on the *x*‐axis in following order: Bird, lizard, crab and colors represent the morphotype as indicated on the legend.

**FIGURE A2:**
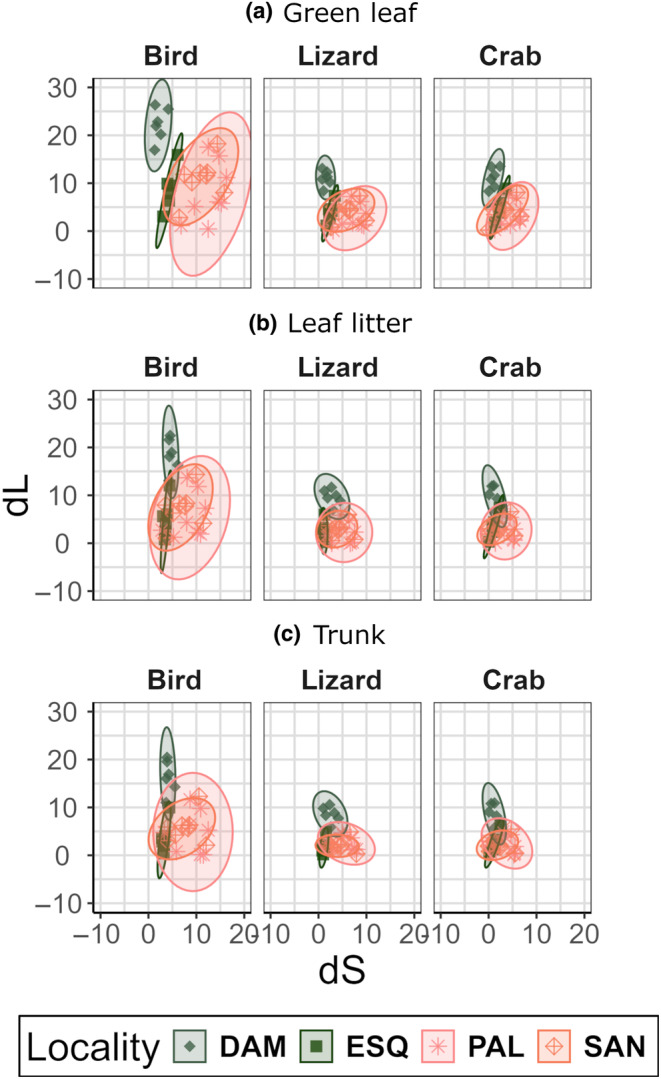
Overall conspicuousness of *Oophaga granulifera* frogs represented by visual model estimates of their color Δ*S* and brightness Δ*L* contrasts on different substrates for three different predators. Symbol shapes indicate each of the localities studied with the corresponding 95% confidence ellipses, symbol colors denote the green and red color morphs, see Figure 1 for additional info on the localities. Panel columns display the different modeled predators (from left to right: Bird, lizard, and crab) with panel rows indicating the calculated visual contrast against each substrate types: Green leaf (a); leaf litter (b), and trunk (c).

**FIGURE A3:**
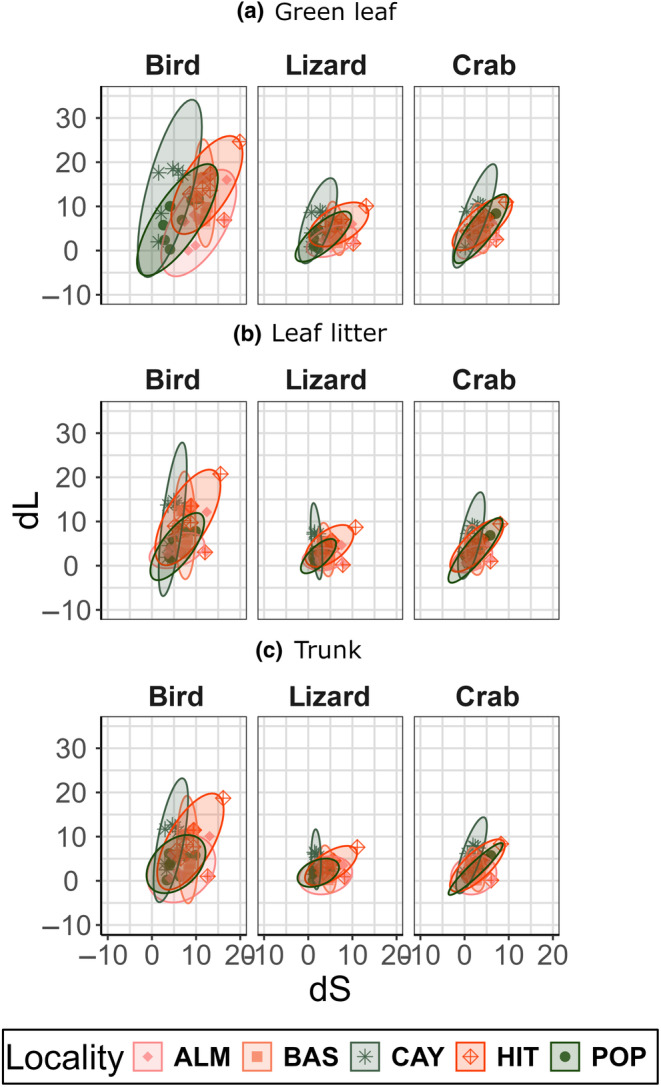
Overall conspicuousness of *Oophaga pumilio* frogs represented by visual model estimates of their color Δ*S* and brightness Δ*L* contrasts on different substrates for three different predators. Symbol shapes indicate each of the localities studied with the corresponding 95% confidence ellipses, symbol colors denote the green and red color morphs, see Figure 1 for additional info on the localities. Panel columns display the different modeled predators (from left to right: Bird, lizard, and crab) with panel rows indicating the calculated visual contrast against each substrate types: Green leaf (a); leaf litter (b), and trunk (c).

In terms of Δ*S*, red and green frogs are more highly differentiated under the visual system of a bird and of a lizard and when contrasted against any of the three substrates (Figure 3; Data S1). Under the visual system of a crab, Δ*S* differences between red and green frogs are not as prominent (Figure 3). In terms of Δ*L*, green *O. granulifera* appear slightly more conspicuous from red frogs (Figure 4). Under the visual system of a bird, Δ*L* is estimated at similar values for green and red frogs (Figure 4, Data S1). Under the visual systems of lizards and birds, green frogs are more differentiated than red frogs (Figure 4). These differences are less strong in *O. pumilio* where frogs of either morph show similar Δ*L* estimates, under all three visual systems and when contrasted against all three substrates (Figure 4). Overall conspicuousness is represented by combining estimated Δ*S* and Δ*L* in a luminance and color contrast space (Appendix 1: Figures A2 and A3). Red *O. pumilio* and *O. granulifera* appear to be more highly conspicuous under the avian visual system and when contrasted to green leaves (Appendix 1: Table A1, A2, A3, Figures A2 and A3). Under the visual system of a lizard, red frogs appear more conspicuous than green frogs against green leaves but not as conspicuous as under the avian visual system. Differences in conspicuousness levels are less strong under the visual system of a crab (Appendix 1: Table A1, Figures A2 and A3).

### 3.2 Effect of predictor variables on response variables

**FIGURE 5:**
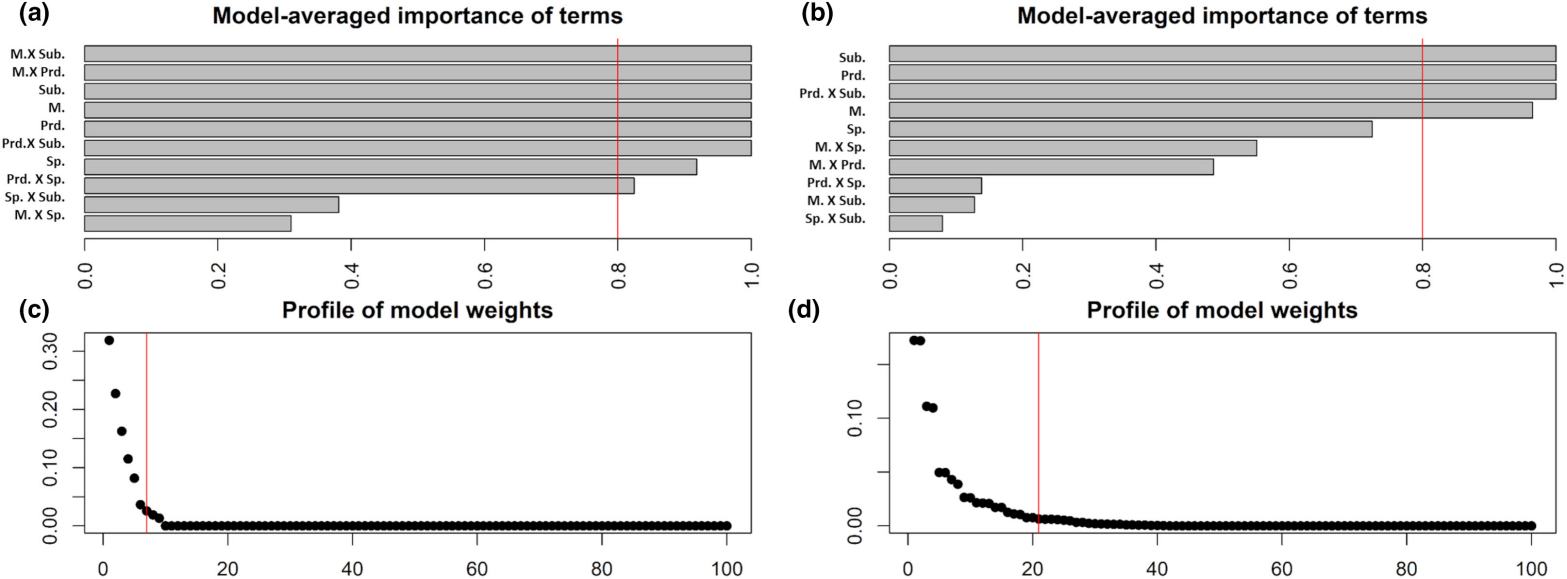
Averaged importance of terms and model ranking of multiple models explaining the variation in color (Δ*S*) and luminance (Δ*L*) contrasts of *Oophaga* frogs by the effects of four predictors (predator, substrate, morph, and frog species) and their interactions. Top row, model‐averaged importance of predictors (the red line indicates the 80% threshold) for the predicted variable Δ*S* (a) and Δ*L* (b). Bottom row: Evidence weight of models of predicted variable, ranked according to their AICc (the vertical red line indicates the 95% evidence weight threshold) for Δ*S* (c) and Δ*L* (d). Abbreviations as in Tables 1 and 2.

We detected no effect of frog species for Δ*S* measurements, after controlling for the effect of other predictors. An effect of species is observed for Δ*L* measurements, after controlling for the effect of other predictors. This result implies that the coloration of the two color morphs studied is equivalent to predators across the two species in terms of color contrast but not of luminance contrast (e.g. differences in amount of light reflected from an object and the background on which it occurs). The top set of models for Δ*S* included 7 models with the most important terms being the four predictors and four interactions (morph x substrate, morph x predator, predator x substrate and predator x species). For Δ*L* the top set included 16 models with substrate, predator, morph, and the predator x substrate interaction being the most important terms (Figure 5, Tables A2 and A3).

**TABLE 1:** Model‐averaged estimates of predictor variables after model selection and averaging for Δ*S*.

Term	Nb models	Im.	L. CI 95%	Up. CI 95%	Est.
**(Intercept)**	**6**	**1**	**4.181**	**6.418**	**5.300**
**M.: Red**	**6**	**1**	**4.571**	**7.272**	**5.922**
**Prd.: Crab**	**6**	**1**	**−3.916**	**−3.090**	**−3.503**
**M.: Red x Prd.: Crab**	**6**	**1**	**−3.623**	**−2.839**	**−3.231**
**M.: Red x Sub.: L.L.**	**6**	**1**	**−3.238**	**−2.450**	**−2.844**
**M.: Red x Sub.: Tr.**	**6**	**1**	**−2.681**	**−1.894**	**−2.288**
**Prd.: Crab x Sub.: L.L.**	**6**	**1**	**0.604**	**1.558**	**1.081**
**Prd.: Crab x Sub: Tr.**	**6**	**1**	**0.566**	**1.520**	**1.043**
**Sub.: Tr.**	**6**	**1**	**−1.022**	**−0.141**	**−0.581**
Sub.: L.L.	6	1	−0.848	0.051	−0.398
M.: Red x Prd.: Liz.	6	1	−0.516	0.269	−0.124
Prd.: Liz.	6	1	−0.535	0.300	−0.118
Prd.: Liz. x Sub.: L.L.	6	1	−0.498	0.456	−0.021
Prd.: Liz. x Sub.: Tr.	6	1	−0.472	0.482	0.005
Sp.: *O. pumilio*	5	0.536	−0.745	1.090	0.173
Prd.: Liz. x Sp.: *O. pumilio*	2	0.131	−0.188	0.111	−0.039
Prd.: Crab x Sp.: *O. pumilio*	2	0.131	−0.151	0.092	−0.029
M.: Red x Sp.: *O. pumilio*	1	0.062	−0.202	0.173	−0.014
Sp.: *O. pumilio* x Sub: L.L.	3	0.308	−0.485	0.245	−0.120
Sp.: *O. pumilio* x Sub: Tr.	3	0.308	−0.425	0.221	−0.102

*Note*: Predictor variables in bold indicate the variables who have a statistically strong effect.

Abbreviations: Est., coefficient estimates; Importance, variable importance; L. CI 95%, lower 95% confidence interval; L.L., leaf litter; Liz., lizard; M., morphotype; Nb models, number of models with the variable; Prd., predator; Sp., species; Sub., substrate; Tr., trunk; Up. CI 95%, upper 95% confidence interval.

**TABLE 2:** Model‐averaged estimates of predictor variables after model selection and averaging for Δ*L*.

Term	Nb models	Imp.	L. CI 95%	Up. CI 95%	Est.
**(Intercept)**	**9**	**1**	**13.21**	**17.391**	**15.3**
**Prd.: Crab**	**9**	**1**	**−7.433**	**−5.756**	**−6.594**
**Prd.: Liz.**	**9**	**1**	**−8.323**	**−6.74**	**−7.532**
**M.: Red**	**9**	**1**	**−6.301**	**−0.427**	**−3.364**
**Sub.: L.L**	**9**	**1**	**−3.913**	**−2.626**	**−3.269**
**Sub.: Tr.**	**9**	**1**	**−5.525**	**−4.172**	**−4.848**
**Sp.: *O. pumilio***	**9**	**1**	**−6.943**	**−1.041**	**−3.992**
**Prd: Crab x Sub.: L.L.**	**9**	**1**	**1.075**	**2.738**	**1.907**
**Prd.: Liz. x Sub.: L.L**	**9**	**1**	**1.249**	**2.912**	**2.08**
**Prd.: Crab x Sub.: Tr.**	**9**	**1**	**1.828**	**3.49**	**2.659**
**Prd.: Crab x Sp.: *O. pumilio***	**9**	**1**	**0.545**	**1.927**	**1.236**
**Prd.: Lizard x Sp.: *O. pumilio***	**9**	**1**	**0.376**	**1.756**	**1.066**
M.: Red x Prd.: Crab	6	0.886	−1.573	0.039	0.767
M.: Red x Prd.: Liz.	6	0.886	−0.61	0.612	0.001
M.: Red x Sp.: *O. pumilio*	6	0.780	−1.282	6.994	2.856
Sp.: *O. pumilio* x Sub.: L.L.	4	0.257	−0.239	0.384	0.073
Sp.: *O. pumilio* x Sub.: Tr.	4	0.257	−0.325	0.594	0.134
M.: Red x Sub.: L.L.	2	0.084	−0.074	0.063	0.005
M.: Red x Sub.: Tr.	2	0.084	−0.112	0.079	0.017

*Note*: Predictor variables in bold indicate the variables who have a statistically strong effect.

Abbreviations: Est., coefficient estimates; Importance, variable importance; L. CI 95%, lower 95% confidence interval; L.L., leaf litter; Liz., lizard; M., morphotype; Nb models, number of models with the variable; Prd., predator; Sp., species; Sub., substrate; Tr., trunk; Up. CI 95%, upper 95% confidence interval.

In Table 1, model averaged estimates of Δ*S* are shown as estimated when *O. granulifera*, bird, green and green leaf are the reference of each predictor variable (species, predator, morph, substrate). Model averaged estimates of Δ*S*, indicate that, after controlling for the effects of the other predictors, red frogs are more color conspicuous than green frogs. Likewise, frogs are more color conspicuous to birds and lizards than to crabs with no strong differences between birds and lizards. Regarding the substrate, frogs are similar in color conspicuousness against green leaves and leaf litter, but less color conspicuous against trunks. Interactions between morph and substrates indicate that red frogs are less color conspicuous when perching on leaf litter or trunks as compared to green leaves. The interaction between predator and substrate has a strong effect on Δ*S*, for example: for a crab, color contrast of the frogs is greater when observed on leaf litter or trunks than on green leaves. Finally, the interaction of morph and predator has also a strong effect on Δ*S*, for example: red frogs appear less color conspicuous to crabs as compared to the other two predators (Table 1).

In Table 2, model averaged estimates of Δ*L* are shown as estimated when *O. granulifera*, bird, green and green leaf are the reference of each predictor variable (species, predator, morph, substrate). Model averaged estimates of Δ*L* indicate that birds are able to perceive higher luminance contrasts between frogs and substrate than crabs and lizards. An effect of morph on Δ*L* shows red frogs to be less conspicuous, in terms of luminance, after controlling for the effect of other predictors. A frog seems to appear more conspicuous in terms of luminance, when contrasted against a green leaf than against a trunk or leaf litter. An effect of species on Δ*L*, indicates that *O. granulifera* appear more conspicuous in terms of luminance contrast. Interactions between species and predator have an effect on Δ*L*, where a higher Δ*L* of *O. pumilio* frogs under the visual systems of crabs and lizards is observed. Interactions between predator and substrate has an effect on Δ*L*. For example, Δ*L* is estimated higher under the visual system of birds and when frogs are perching on leaf litter or trunks (Table 2).

## 4 DISCUSSION

Our results show that color contrasts in these two *Oophaga* species differ due to the effects of the morphotype, predator and substrate type but not due to inter‐specific differences between *O. pumilio* and *O. granulifera*. Luminance contrasts, however, differ due to the effects of predator, morphotype, substrate but also between the two frog species. The fact that we also found important interactions between the predictors of Δ*S* (between morph, substrate and predator) and Δ*L* (between substrate and predator) values suggests that specific combinations of these three factors can result in increased conspicuousness of these frog color phenotypes (see Tables 1 and 2, Figures 3, 4 and 5).

Under the visual system of an avian UV‐sensitive predator, color and luminance contrasts between frogs and their substrate are more pronounced than those between crabs and their substrate, suggesting that birds are better equipped to visually distinguish frogs from their surroundings. Thus, color signals seem to be perceived better by bird predators, as has been previously suggested in similar studies for *O. pumilio* (Maan & Cummings, 2012; Pröhl & Ostrowski, 2011; Siddiqi et al., 2004). Additionally, no strong statistical differences are observed in estimated color contrasts between birds and lizards, indicating that both predators have a similar ability to detect the frogs against the substrate. In both predators' conceptual space, red and green morphs occupy opposite spaces, showing a strong ability to differentiate between red and green frogs. This shows that lizards perceive color signals similar to birds, suggesting them as an important predator system and highlight the importance of increasing the number of model species in visual ecology research.

In *O. pumilio*, predation pressure has been suggested to promote color divergence and red morphotypes of *O. pumilio* have been found to be less palatable than the green morphotypes on Bocas del Toro (Maan & Cummings, 2012). Predation experiments on different *O. pumilio* morphs from Bocas del Toro and on the ancestral mainland morphotype showed that local cryptically colored (green) morphs were attacked more often than non‐local red and brown clay models (Hegna et al., 2013). Additionally, birds appear to attack cryptically colored clay models of *O. pumilio* more frequently than aposematically colored clay models in several localities (Paluh et al., 2014; Saporito et al., 2006). Birds recognize unpalatable prey by their conspicuousness and through aversion learning, where an aposematic signal (like red coloration) is associated with a feeling of distastefulness and thus is avoided (Gittleman & Harvey, 1980). Birds have been suggested as the main predators of *O. pumilio* frogs (Dreher et al., 2015) and our results align with these findings. Additionally, we provide evidence that lizards perceive aposematic signals equally to birds. Attack or predation rates of *O. pumilio* by non‐avian predators are less common (Dreher et al., 2015), however, lizards could be an important predator in some localities. Additional evidence on attack and predation rate of frogs by lizards can show whether shifts in prey conspicuousness among localities is related to variations in predator composition.

Contrary to what is observed in *O. pumilio*, in *O. granulifera*, more brightly colored morphs (red aposematic morphs) are less toxic than cryptic morphs (green and yellow) (Wang, 2011). In *O. granulifera*, predation experiments showed a higher tendency of birds to avoid clay models with local color, whether that was red, intermediate or green. Lizards preferred attacking red colored clay models in *O. granulifera* populations (Willink et al., 2014). The higher attack rate on red colored clay models from lizards could be related to their ability of detecting the frogs and the lack of an aversive association with red in *O. granulifera*.

Luminance contrast is an additional component of aposematic coloration and can be effective as a warning signal (Prudic et al., 2007). Additionally, it can be of importance for colorblind predators and could promote conspicuousness in opposite directions than color contrast. In *O. pumilio*, luminance contrasts between red and green frogs are similar. It has been previously reported that in *O. pumilio* luminance contrasts between frogs and substrate are less prominent than color contrasts (Siddiqi et al., 2004). Our results agree and show no strong differentiation between aposematic red and cryptic green frogs in terms of luminance contrast of frogs against background. In *O. granulifera*, luminance contrast follows a different pattern. Conspicuousness of *O. granulifera* has been evaluated before by calculating only color contrast between frog and common background (including leaf litter, buttress roots, mossy surfaces, and green broadleaf plants) (Wang, 2011). In another study, color and luminance contrast of green, red and intermediate *O. granulifera* morphs from different localities showed that red frogs are more conspicuous in both color and luminance contrasts (Willink et al., 2013). In our study, we found that the less conspicuous green *O. granulifera* frogs in terms of color contrast appear more conspicuous in terms of luminance contrast, with some variation between localities. Additionally, an effect of interactions between predator and species on Δ*L* is observed, where *O. granulifera* appear to be higher in luminance contrast than *O. pumilio*. This could be the result of different predation pressures on each locality and each species that in the case of *O. granulifera* promotes higher luminance contrasts as a result of an achromatic (e.g, luminance) aposematic signal rising from green *O. granulifera* frogs.

Theory predicts that toxicity evolves in tandem with bright coloration, as in several aposematic species, higher conspicuousness is connected to higher unpalatability (Blount et al., 2009; Maan & Cummings, 2012). This is observed in *O. pumilio*, where brighter, more conspicuous morphotypes are more unpalatable (Maan & Cummings, 2012). However, unpalatability in aposematic and cryptic morphs of *O. granulifera* follows a contradictory trend (Wang, 2011). The overall higher luminance contrasts of green *O. granulifera* compared to red *O. granulifera*, could indicate the presence of an aposematic signal in some green *O. granulifera* populations. In *O. pumilio*, where red frogs are more toxic, such an aposematic signal observed in green frogs may not be needed due to them being more palatable than the red ones. This may suggest that the color signal alone is a stronger aposematic signal in this case.

These observed differences in conspicuousness between morphs, may further be linked to the varying conditions in each locality. Predator pressure and mate selection may drive color and luminance variation in species occupying different habitats where light conditions and background are varying (Dugas & Franssen, 2011; Galeotti et al., 2003; Rosenblum, 2006; Rudh et al., 2011). Current natural conditions of each locality can further influence the appearance of frogs occupying this locality to predators and they can appear slightly more or less conspicuous than other similar morphotypes, promoting different level of color and luminance contrast between localities. For example, substrate is playing a key role in how conspicuous prey appears to potential predators (Defrize et al., 2010; Prudic et al., 2007) and a strong effect of substrate on overall conspicuousness is also observed in our study. In Neotropical birds, background selection plays a role in advertising their color signals for mate attraction (Endler & Thery, 1996). Substrate selection could be another antipredatory behavior and need to be further explored.

In addition to these environmental factors, differences in conspicuousness between localities may also be linked to the varying alkaloid profiles prevailing in each area. The alkaloid content in *O. pumilio* and *O. granulifera* is related to the alkaloid‐containing arthropods in their diet. Alkaloid profiles differ between populations in quantity and/or structure due to the differences in alkaloid‐containing arthropod availability in each locality (Jeckel et al., 2022; Prates et al., 2019; Saporito, Donnelly, et al., 2007; Saporito et al., 2004, 2006).

These alkaloids in *O. pumilio* frogs have been shown to be unpalatable to various organisms, including arthropods (ants, fruit fly), laboratory mice and domestic chickens (Bolton et al., 2017; Darst et al., 2006; Darst & Cummings, 2006; Stuckert et al., 2014). Although alkaloids can be particularly toxic for certain arthropods (fire ant, *Solenopsis invicta*), they are distasteful for domestic chickens and mostly result in rejection and beak wiping (Bolton et al., 2017; Darst et al., 2006; Darst & Cummings, 2006; Stuckert et al., 2014). Not much is known on how lizards and crabs respond to *Oophaga*‐related toxins but there is evidence that alkaloids such as the ones found in fire ants have an aversive effect on *Anolis* lizards (Boronow & Langkilde, 2010).

The predator's response to such alkaloids play a critical role in the effectiveness of the aposematic, conspicuous signal. The chemical defenses of the aposematic prey will then result in the promotion of the aposematic signal through aversion learning of the predator population (Ham et al., 2006; Lindström et al., 2001; Prudic et al., 2007; Skelhorn & Rowe, 2006). The crucial point of an aposematic signal to be recognized and avoided is determined by the associative strength of the signal and the chemical defense (Aronsson & Gamberale‐Stille, 2013; Halpin et al., 2008).

Birds have been suggested as important predators of *Oophaga* frogs because of their ability to perceive aposematic signals, their avoidance of non local and conspicuous prey (avoidance of aposematic or foreign signal) and the evidence for alkaloid aversion (Bolton et al., 2017; Dreher et al., 2015; Maan & Cummings, 2012). The avoidance of non local coloration and conspicuous morphs (avoidance of unknown signal and avoidance of warning signal), suggests that predation pressure played a role in the divergence of color morphs, at least in Bocas del Toro, and could be the case in other *Oophaga* frogs. Diurnal lizards are likely to exert strong predation pressure, due to their ability to perceive the aposematic signal, however, more information on empirical predation rates are needed to answer that.

Our analysis comprises the first comparison of visual conspicuousness between extreme morphotypes of *O. pumilio* and *O. granulifera* and provides a first estimation of how similar equivalent morphotypes of aposematic red and cryptic green coloration of the two species are. *Anolis* lizards seem to have a similar ability to birds in observing visual differences between red and green frogs, while crabs seem to be less able to perceive those visual differences. Color contrasts of frogs and substrate appear similar between the two species, however luminance contrasts show differences as *O. granulifera* appear brighter than red frogs while the opposite is true for *O. pumilio*. Such a signal could be related to opposite toxicity patterns and differences in predation pressure upon the two species and among localities of the two species.

## AUTHOR CONTRIBUTIONS

**Vasiliki Mantzana‐Oikonomaki:** Conceptualization (equal); data curation (equal); formal analysis (equal); investigation (equal); methodology (equal); visualization (lead); writing – original draft (equal); writing – review and editing (equal). **Ariel Rodríguez:** Conceptualization (equal); data curation (equal); formal analysis (equal); funding acquisition (lead); investigation (equal); methodology (equal); resources (equal); supervision (equal); visualization (supporting); writing – original draft (equal); writing – review and editing (equal). **Giselle Castillo‐Tamayo:** Conceptualization (equal); funding acquisition (lead); writing – review and editing (supporting). **Roberto Ibáñez:** Conceptualization (supporting); data curation (equal); investigation (supporting); resources (equal); writing – review and editing (supporting). **Heike Pröhl:** Conceptualization (equal); funding acquisition (lead); investigation (equal); project administration (lead); resources (equal); supervision (equal); writing – review and editing (supporting).

## CONFLICT OF INTEREST STATEMENT

The authors declare that the research was conducted in the absence of any commercial or financial relationships that could be construed as a potential conflict of interest.

## Supporting information


Data S1.

## Data Availability

Raw data on spectral measurements generated during this project alongside codes generated for the analysis of these data and final outputs are available at the github repository associated with this project which is open to the public (github.com/BillieMantzOik/COLORVAR).

## APPENDIX 1

**TABLE A1:** Average measurements of calculated Δ*S* and *ΔL* for all *Oophaga granulifera* (left side of table) and *O. pumilio* (right side of table) frogs collected in each study locality and against three types of natural substrate (Green leaf, leaf litter, trunk).

Loc.	Sub.	dS	Dssd	dL	Dlsd	Prd	Sp	m	Loc.	Sub.	dS	Dssd	dL	Dlsd	Prd	Sp	m
DAM	Gr.L.	2.078	0.983	18.821	7.068	Bird	*O. granulifera*	Green	CAY	Gr. L.	3.626	2.319	10.765	7.880	Bird	*O. pumilio*	Green
DAM	L.L.	4.649	0.694	14.945	7.068				CAY	L. L.	4.690	1.073	8.203	6.279			
DAM	Tr.	4.056	0.721	14.689	6.826				CAY	Tr.	4.229	1.275	7.691	4.613			
DAM	Gr.L.	0.763	0.805	9.368	3.788	Crab			CAY	Gr. L.	1.948	1.519	6.071	4.287	Crab		
DAM	L.L.	1.248	0.721	7.870	3.788				CAY	L. L.	1.312	0.909	4.763	4.043			
DAM	Tr.	1.146	0.688	6.761	3.788				CAY	Tr.	1.313	1.042	4.353	3.229			
DAM	Gr.L.	2.439	1.035	9.524	3.538	Lizard			CAY	Gr. L.	3.749	2.705	5.694	4.052	Lizard		
DAM	L.L.	4.903	1.397	4.903	3.538				CAY	L. L.	3.817	0.648	4.355	3.561			
DAM	Tr.	4.356	1.346	4.356	3.538				CAY	Tr.	3.535	0.933	4.056	2.719			
ESQ	Gr.L.	6.670	4.241	10.638	5.033	Bird			POP	Gr. L.	6.322	3.205	8.174	5.430	Bird		
ESQ	L.L.	5.782	3.639	5.782	4.702				POP	L. L.	6.442	2.351	5.636	3.858			
ESQ	Tr.	5.571	4.185	5.571	4.141				POP	Tr.	6.153	2.542	4.654	3.380			
ESQ	Gr.L.	3.900	2.909	6.304	3.066	Crab			POP	Gr. L.	3.997	2.108	5.603	3.323	Crab		
ESQ	L.L.	2.604	2.909	4.806	3.066				POP	L. L.	2.701	2.108	4.249	3.109			
ESQ	Tr.	6.199	4.202	5.094	4.181				POP	Tr.	2.859	2.108	3.535	2.649			
ESQ	Gr.L.	7.180	5.036	5.911	2.656	Lizard			POP	Gr. L.	7.340	4.015	4.588	2.959	Lizard		
ESQ	L.L.	5.145	4.300	4.178	2.656				POP	L. L.	5.461	3.034	3.314	2.350			
ESQ	Tr.	6.250	4.189	3.349	2.348				POP	Tr.	5.478	3.325	2.796	1.898			
PAL	Gr.L.	12.593	3.153	7.885	6.335	Bird		Red	ALM	Gr. L.	11.073	3.530	6.605	4.851	Bird		Red
PAL	L.L.	8.277	3.054	5.656	4.790				ALM	L. L.	6.803	3.431	4.377	3.186			
PAL	Tr.	8.841	3.087	4.861	4.365				ALM	Tr.	7.357	3.461	3.353	3.349			
PAL	Gr.L.	4.748	1.978	3.328	2.671	Crab			ALM	Gr. L.	3.602	2.113	2.564	1.801	Crab		
PAL	L.L.	3.458	1.966	2.579	1.966				ALM	L. L.	2.306	2.113	1.816	1.408			
PAL	Tr.	3.610	1.978	2.447	1.978				ALM	Tr.	2.464	2.113	1.525	1.640			
PAL	Gr.L.	12.368	4.272	3.854	3.013	Lizard			ALM	Gr. L.	10.102	4.674	3.069	2.248	Lizard		
PAL	L.L.	8.703	3.789	2.872	2.311				ALM	L. L.	6.673	4.236	2.123	1.450			
PAL	Tr.	9.191	3.935	2.441	2.119				ALM	Tr.	7.109	4.346	1.605	1.572			
SAN	Gr.L.	10.871	3.211	10.887	4.349	Bird			BAS	Gr. L.	11.812	0.955	12.973	4.225	Bird		
SAN	L.L.	6.838	2.746	7.273	3.827				BAS	L. L.	7.373	0.925	9.097	4.225			
SAN	Tr.	7.341	2.851	5.737	3.075				BAS	Tr.	7.978	0.931	7.050	4.225			
SAN	Gr.L.	3.148	2.300	4.172	2.198	Crab			BAS	Gr. L.	3.655	0.751	5.030	1.892	Crab		
SAN	L.L.	2.226	1.893	3.049	1.893				BAS	L. L.	2.359	0.751	3.533	1.892			
SAN	Tr.	2.305	1.967	2.217	1.967				BAS	Tr.	2.517	0.751	2.594	1.651			
SAN	Gr.L.	9.688	4.492	5.080	2.218	Lizard			BAS	Gr. L.	10.728	1.493	5.803	2.064	Lizard		
SAN	L.L.	6.786	3.401	3.549	3.401				BAS	L. L.	6.908	1.359	4.070	2.064			
SAN	Tr.	7.122	3.625	5.377	1.498				BAS	Tr.	7.454	1.389	3.060	2.019			
									HIT	Gr. L.	13.080	3.531	14.726	5.240	Bird		
									HIT	L. L.	8.865	3.342	10.850	5.240			
									HIT	Tr.	9.416	3.380	8.803	5.240			
									HIT	Gr. L.	4.535	2.805	6.001	2.637	Crab		
									HIT	L. L.	3.408	2.566	4.503	2.637			
									HIT	Tr.	3.527	2.616	3.424	2.593			
									HIT	Gr. L.	12.661	5.446	6.932	2.556	Lizard		
									HIT	L. L.	9.266	4.818	5.199	2.556			
									HIT	Tr.	9.717	4.948	4.163	2.556			

*Note*: Standard deviation is also estimated for Δ*S* (dssd) and *ΔL* (dlsd).

Abbreviations: dL., *ΔL*; dlsd., *ΔL* standard deviation; dS, Δ*S*; dssd, Δ*S* standard deviation; Loc., locality; m., morphotype; Prd., predator; Sp., species; Sub., substrate.

**TABLE A2:** Results of the model selection analysis of the relationship between predictor variables (predator, substrate, morph, and species) and the color contrast (dS) of *Oophaga* frogs.

Rank	Model	Aicc	ΔAIC	Weights	ER	C.Weight
**1**	*dS ~ 1 + Prd. + M. + Sub. + + Sp. + M. x Prd. + Sub*. × *Prd. + Sub*. × *M*. *+ Sp. x Prd.*	1662.096	0.00	0.318	1.00	0.319
**2**	*dS ~ 1 + Prd. + M. + Sub. +* Sp. *+ M.:Prd. + Sub*. × *Prd. + Sub*. × *M*. *+ Sp. x Prd. + Sp. x Sub.*	1662.772	0.676	0.227	1.402	0.546
**3**	*dS ~ 1 + Prd. + M. + Sub. +* Sp. *+ M*. × *Prd. + Sub*. × *Prd. + Sub*. × *M. +* Sp. × *Prd*. *+ Sp x M.*	1663.440	1.345	0.163	1.959	0.709
**4**	*dS ~ 1 + Prd. + M. + Sub. +* Sp. *+ M. × Prd. + Sub. × Prd. + Sub. × M. +* *Sp. x Prd. + Sp. x M. +* Sp. × Sub.	1664.132	2.036	0.115	2.768	0.824
**5**	*dS ~ 1 + Prd. + M. + Sub. +* * M*. × *Prd. + Sub*. × *Prd. + Sub*. × *M.*	1664.814	2.718	0.082	3.892	0.906
**6**	*dS ~ 1 + Prd. + M. + Sub. +* Sp. *+ M*. × *Prd. + Sub*. × *Prd. + Sub*. × *M.*	1666.434	4.338	0.036	8.751	0.943
**7**	*dS ~ 1 + Prd. + M. + Sub. +* Sp. *+ M*. × *Prd. + Sub*. × *Prd. + Sub*. × *M. +* Sp. × *Sub.*	1667.140	5.044	0.025	12.454	0.968

*Note*: Models are ranked by their corrected Akaike Information Criteria (AICc), indicating the differences between a given model and the best fitting model (ΔAICc), the model weight, and the ER which is the ratio between the weights of the best model and competing models. Only the models in the high confidence set (summing up to 95% of the evidence weight) are shown.

Abbreviations: M., morphotype; Prd., predator; Sp., species; Sub., substrate.

**TABLE A3:** Results of the model selection analysis of the relationship between predictor variables (predator, substrate, morph, and species) and the luminance contrast (dL) of *Oophaga* frogs.

Rank	Model	Aicc	ΔAIC	Weights	ER	C.Weight
1	*dL ~ 1 + Prd. + M. + Sub. + Sp. + Sub. × Prd. + Sp. × M*.	2611.3194	0.000	0.172	1.000	0.172
2	*dL ~ 1 + Prd. + M. + Sub. + Sp. + M. × Prd. + Sub. × Prd. + Sp. × M*.	2611.3224	0.003	0.172	1.002	0.344
3	*dL ~ 1 + Prd. + M. + Sub. + M. × Prd. + Sub. × Prd*.	2612.1966	0.877	0.111	1.551	0.456
4	*dL ~ 1 + Prd. + M. + Sub. + Sub. × Prd*.	2612.2231	0.904	0.110	1.571	0.565
5	*dL ~ 1 + Prd. + M. + Sub. + Sp. + M. × Prd. + Sub. × Prd*.	2613.8093	2.490	0.050	3.473	0.615
6	*dL ~ 1 + Prd. + M. + Sub. + Sp. + Sub. × Prd*.	2613.8210	2.502	0.049	3.493	0.664
7	*dL ~ 1 + Prd. + M. + Sub. + Sp. + M. × Prd. + Sub. × Prd. + Sp. × Prd. + Sp. × M*.	2614.0984	2.779	0.043	4.013	0.707
8	*dL ~ 1 + Prd. + M. + Sub. + Sp. + Sub. × Prd. + Sp. × Prd. + Sp. × M*.	2614.3112	2.992	0.039	4.464	0.746
9	*dL ~ 1 + Prd. + M. + Sub. + Sp. + Sub. × Prd. + Sub. × M. + Sp. × M*.	2615.0742	3.755	0.026	6.537	0.772
10	*dL ~ 1 + Prd. + M. + Sub. + Sp. + M. × Prd. + Sub. × Prd. + Sub. × M. + Sp. × M*.	2615.1033	3.784	0.026	6.632	0.798
11	*dL ~ 1 + Prd. + M. + Sub. + Sp. + Sub. × Prd. + Sp. × M. + Sp. × Sub*.	2615.4876	4.168	0.021	8.038	0.820
12	*dL ~ 1 + Prd. + M. + Sub. + Sp. + M. × Prd. + Sub. × Prd. + Sp. × M. + Sp. × Sub*.	2615.5201	4.201	0.021	8.169	0.841
13	*dL ~ 1 + Prd. + Sub. + Sub. × Prd*.	2615.5587	4.239	0.021	8.328	0.862
14	*dL ~ 1 + Prd. + M. + Sub. + M. × Prd. + Sub. × Prd. + Sub. × M*.	2615.9476	4.628	0.017	10.116	0.879
15	*dL ~ 1 + Prd. + M. + Sub. + Sub. × Prd. + Sub. × M*.	2615.9484	4.629	0.017	10.120	0.896
16	*dL ~ 1 + Prd. + M. + Sub. + Sp. + M. × Prd. + Sub. × Prd. + Sp. × Prd*.	2616.5702	5.251	0.012	13.811	0.908
17	*dL ~ 1 + Prd. + M. + Sub. + Sp. + Sub. × Prd. + Sp. × Prd*.	2616.7981	5.479	0.011137	15.477	0.919
18	*dL ~ 1 + Prd. + Sub. + Sp. + Sub. × Prd*.	2616.9167	5.597	0.010496	16.423	0.930
19	*dL ~ 1 + Prd. + M. + Sub. + Sp. + Sub. × Prd. + Sub. × M*.	2617.5611	6.242	0.007605	22.665	0.937
20	*dL ~ 1 + Prd. + M. + Sub. + Sp. + M. × Prd. + Sub. × Prd. + Sub. × M*.	2617.5752	6.256	0.007552	22.826	0.945
21	*dL ~ 1 + Prd. + M. + Sub. + Sp. + M. × Prd. + Sub. × Prd. + Sub. × M. + Sp. × Prd. + Sp. × M*.	2617.9081	6.589	0.006394	26.960	0.951

*Note*: Models are ranked by their corrected Akaike Information Criteria (AICc), indicating the differences between a given model and the best fitting model (ΔAICc), the model weight, and the ER which is the ratio between the weights of the best model and competing models. Only the models in the high confidence set (summing up to 95% of the evidence weight) are shown.

Abbreviations: M., morphotype; Prd., predator; Sp., species; Sub., substrate.
